# Factors associated with prior pregnancy loss among multigravid women: a retrospective machine learning analysis from an African population cohort

**DOI:** 10.3389/fgwh.2026.1803064

**Published:** 2026-09-17

**Authors:** Patricia Nabisubi, Timothy Mwanje Kintu, Mugume Twinamatsiko Atwine, James Bumba, Lawrence Muwonge, Herbert Agasi, Davis Kiberu, Julius Okwir, Fredrick Elishama Kakembo, Daudi Jjingo, Annettee Nakimuli, Ronald Galiwango

**Affiliations:** 1 The African Center of Excellence in Bioinformatics and Data Intensive Sciences, Kampala, Uganda; 2 The Infectious Diseases Institute, Makerere University, Kampala, Uganda; 3 Department of Computer Science, College of Computing and Information Sciences, Makerere University, Kampala, Uganda; 4 Department of Obstetrics and Gynaecology, Makerere University and Mulago National Referral Hospital, Kampala, Uganda

**Keywords:** abortion, African population, machine learning, maternal health, pregnancy loss

## Abstract

**Background:**

Pregnancy loss remains a significant challenge in obstetric care, with traditional risk assessment methods, such as clinical history and radiological investigations, often lacking precision in predicting outcomes. This study applied machine learning (ML) to develop a robust model for identifying factors associated with pregnancy loss, with the goal of highlighting candidate predictors for further investigation and validation in future studies in African populations.

**Methods:**

The study utilized sociodemographic, clinical and laboratory variables collected at baseline from an ongoing cohort study titled “Future maternal cardiovascular health after pre-eclampsia in an indigenous African population”. A total of 1,017 pregnant women attending Mulago National Referral Hospital in Kampala, Uganda were enrolled between 2019 and 2021. However, the outcome question was not administered to women on their first pregnancy, limiting the modeling population to multigravid women (*n* = 655). Advanced ML tools in R and Python were employed to process the de-identified data, including rigorous data cleaning, feature selection, and handling of missing values. Several ML classifiers were trained and evaluated using cross-validation techniques and the training-test split approach, with performance assessed using Matthew's Correlation Coefficient (MCC), precision, recall and F1 score.

**Results:**

Random Forest achieved the highest discrimination (MCC 0.36, 95% CI 0.25–0.47; ROC-AUC 0.74, 95% CI 0.63–0.83), followed closely by CatBoost (MCC 0.35, 95% CI 0.18–0.46; ROC-AUC 0.71, 95% CI 0.61–0.77); confidence intervals for the two ensemble models overlapped substantially. Both outperformed logistic regression (MCC 0.22, 95% CI 0.08–0.35). Across both tree-based models and both SHAP and permutation-based importance methods, the number of pregnancies carried past seven months, maternal age, and maternal education ranked as the leading correlates. Subgroup analysis showed broadly consistent discrimination across reliable age, education, and maternal tribe.

**Conclusion:**

A few sociodemographic and obstetric history factors are statistically associated with a documented history of pregnancy loss among multigravid women in this Ugandan cohort. Because the outcome reflects reproductive history rather than the result of an observed pregnancy, these findings should be read as hypothesis-generating groundwork for future prospective, externally validated risk-prediction research rather than as a clinical tool for identifying women at risk of a future miscarriage.

## Background

Maternal and child health in sub-Saharan Africa (sSA) remains a significant public health concern, characterized by high rates of maternal and child mortality and morbidity. Among these issues is the high burden of miscarriages, a distressing outcome affecting a considerable number of pregnancies (1). According to the World Health Organization (WHO), a miscarriage is the spontaneous loss of a fetus before the age of viability. In Uganda, approximately one-third of women experience at least one miscarriage or stillbirth during their lifetime, with significant predictors including age and non-attendance at antenatal care (ANC) (2). Miscarriage not only brings emotional distress but also poses risks to postpartum health, correlating with increased postpartum mortality (3).

Efforts to mitigate the burden of miscarriage have included improving access to quality ANC, promoting maternal health education, and integrating routine screenings for high-risk pregnancies (4). Despite these efforts, challenges in access to care for timely identification of high-risk pregnancies still exist. Additionally, traditional methods of predicting miscarriage, primarily based on clinical symptoms and maternal history, have limited accuracy. As such, there's a need for innovative approaches to enhance the prediction and early identification of women at high risk of miscarriage (5). ML techniques offer a promising solution by analyzing large datasets to identify subtle patterns and interactions among various risk factors (6).

Literature on the use of ML to predict various pregnancy outcomes has highlighted the versatility and potential of ML applications, from predicting the mode of childbirth to identifying potential complications during pregnancy (7). The current study sought to build on this evidence base on the use of ML models in obstetric populations by by applying ML methods to identify demographic, clinical, and pregnancy-related factors associated with a history of pregnancy loss among women in Uganda. Using routinely collected antenatal care data, we aimed to characterize the variables most strongly associated with prior pregnancy loss and to develop an exploratory prediction model capable of distinguishing women with and without a history of pregnancy loss. The findings are intended to generate hypotheses regarding potentially important predictors of pregnancy loss and to inform future prospective studies evaluating their utility for risk prediction and targeted maternal health interventions.

## Methods

### Data source

This study utilized existing de-identified, baseline data derived from a study titled “Future maternal cardiovascular health after pre-eclampsia in an indigenous African population”. The dataset comprises a total of 1,017 records representing women who were under observation during the primary study (8).

In this original study (8), structured interviews, surveys, medical examinations, laboratory tests (e.g., for HIV status), and medical records were the primary sources of the collected data. Socioeconomic variables, such as occupation and financial class or status, were self-reported and provided insights into the mother's economic well-being. Additional data in the study was collected through questionnaires administered by trained researchers or healthcare professionals. These questionnaires were designed to gather specific information and were administered professionally. This approach helps ensure that the data collected is accurate and consistent. Trained personnel followed established procedures, clarified questions for participants, and maintained data quality. Researchers also oversaw the process of upholding privacy and confidentiality.

The key variables and factors relevant to the current study encompass various demographic and health-related attributes. These include the mother's age, religious affiliation, educational level, marital status, tribal or ethnic background of both the mother and father, maternal grandparents' backgrounds, paternal grandparents' backgrounds, smoking history, alcohol consumption habits, blood pressure readings, history of stroke, HIV status, family history of diabetes, family history of preeclampsia, family history of hypertension, type of pregnancy, pregnancy order, total number of pregnancies, occurrences of abortions or ectopic pregnancies, pregnancy duration, condom usage, number of sexual partners, history of infertility, gestational age, and various socioeconomic factors such as occupation and financial status or class. These variables collectively constitute the core components of the dataset and serve as essential elements for the study's analytical and research purposes.

### Data preparation

Before processing, the initial dataset had 1,017 rows and 170 columns. The data was processed using the R programming language (version 4.3.1) and Rstudio (version 2022.07.1). The packages tidyverse (version 2.0.0) and DataExplorer (version 0.8.3) were used for processing the deidentified data. Duplicate rows, which were identified by duplicate study IDs, were removed from the data set. Additionally, variables that needed labels or were deemed uninformative in predicting the study outcome, based on a review of current literature and expert review, were eliminated, significantly reducing the number available from 170 to 33. Adjustments were made to certain variables; specifically, subcategories of variables such as pre-eclampsia, religious affiliation, and tribe were reduced by merging similar categories. Values representing different religious affiliations were merged into Christians, Moslems, and Others. Multiple tribes were merged based on their geographical location in Uganda (Northern, Western, Eastern, or Southern tribes). Further processing involved the removal of variables with over 90% missing values, resulting in a final dataset containing 21 columns and 1,006 rows.

### Missing data

Residual missingness after this correction was under 2.2% per variable and was imputed using Multiple Imputation by Chained Equations (MICE) (9), which requires data to be Missing at Random (MAR): a condition satisfied by this residual block but not by the structural block it replaces.

### Outcome definition

The outcome of interest was a combined count of prior pregnancy losses, which included abortions, and ectopic pregnancies. This field was blank for women on their first pregnancy, identified via the pregnancy field, because the corresponding question was not administered to them. One case with a genuinely indeterminate history was excluded rather than imputed. All those who indicated having had at least one miscarriage, induced abortion, or ectopic pregnancy were classified as having the outcome (a documented history of pregnancy loss). We combined these three events into a single outcome because, despite differing in mechanism and intent, all three share the same defining endpoint: the pregnancy does not continue to a live birth. Grouping them this way follows prior epidemiological work on pregnancy loss in this setting, which similarly analyzes miscarriage and stillbirth together as instances of adverse pregnancy outcome rather than modeling each mechanism separately (2).

### Independent variables

Independent variables considered non-modifiable risk factors, including age, religious affiliation, level of education, marital status, maternal and paternal tribe, history of smoking, alcohol use, history of stroke, HIV serostatus, family history of diabetes, pre-eclampsia, or hypertension, number of pregnancies carried past seven months, delivery of a baby weighing less than 2.5 kg or more than 4 kg in a previous pregnancy, history of hypertension in a previous pregnancy, and history of infertility. All variables were feature-encoded to numerical values.

One candidate variable, order_preg (total number of pregnancies), was excluded from the final set of independent variables. This variable is related to the outcome and to No_preg (pregnancies carried past seven months) by a fixed arithmetic identity: order_preg equals No_preg plus the count of prior miscarriages, abortions, and ectopic pregnancies, plus one. The remaining 18 variables, spanning sociodemographic, medical-history, and obstetric-history domains, were retained. We considered only independent variables unrelated to the pregnancy current at the time of the interview, so as to keep the analysis focused on prior reproductive history rather than any characteristic of the pregnancy being observed.

### Study population

The modeling population was restricted to multigravid women, defined as those with at least one prior pregnancy (*n* = 655 of 1,017 total participants). Primigravidae were excluded because the outcome variable is not defined independently of parity for this group: it is deterministically zero for every primigravida by construction, not as a measure of risk. Restricting to multigravidae ensures the outcome reflects a genuinely measured quantity for every participant in the modeling population. Of the 655 multigravid women, 223 had a documented history of pregnancy loss, and 432 did not.

### Model development and validation

Three classifiers were compared: Random Forest (10), CatBoost (11), and a logistic-regression baseline (12). Logistic regression was included, given evidence that machine learning methods do not consistently outperform logistic regression once validation bias is accounted for (13).

Discrimination was primarily summarized using the Matthews Correlation Coefficient (MCC), a threshold-independent measure well suited to imbalanced binary classification because it accounts for all four cells of the confusion matrix (Chicco & Jurman, 2020), alongside ROC-AUC (14), precision, and recall.

Model performance was estimated using repeated stratified k-fold cross-validation(Kohavi, 1995) (5 folds, 5 repeats; 25 total train-and-test rounds) as the primary validation method, with bootstrap-based internal validation (15, 16), (50 resamples, optimism-corrected) as an independent cross-check. An automated check flags disagreement between the two methods exceeding 0.15 MCC, a threshold selected to detect known overfitting behavior in unregularized tree-based ensembles.

For each model, performance is reported using five complementary measures: ROC and precision-recall curves (14, 17), calibration (18), confusion matrices, and decision curve analysis (19), all derived from out-of-fold predictions rather than predictions the model has already seen during training.

### Variable importance

Variable importance was assessed using two complementary methods: SHAP (SHapley Additive exPlanations) (20), which quantifies the direction and magnitude of each independent variable's contribution to individual associations, and permutation importance (10, 21), which quantifies the decrease in cross-validated performance when a variable's values are randomly shuffled. Both methods were computed for the two tree-based models (Random Forest, CatBoost); permutation importance alone was computed for the logistic-regression baseline, since SHAP's tree-based estimator does not apply to a linear model.

### Subgroup performance

Model performance was additionally assessed within subgroups defined by maternal age, education level, and maternal tribal region to characterize whether discrimination is consistent across strata. A subgroup was classified as reliable only if the smaller of its two outcome groups (history of loss vs. no history) contained at least 20 women; subgroups below this threshold are reported as unreliable rather than at face value.

## Results

The final total study population consisted of 655 multigravid women, of whom 223 (34.0%) had a documented history of pregnancy loss (miscarriage, induced abortion, or ectopic pregnancy) and 432 (66.0%) did not. The characteristics of the study population are summarized in Table 1.

**Table 1:** Summary statistics of the study population.

Variable	No (*N* = 432)	Yes (*N* = 223)	Overall (*N* = 655)
**Age**			
Mean (SD)	28.57 (5.26)	29.14 (5.65)	28.76 (5.39)
Median [Min, Max]	28.00 [18.00, 46.00]	29.00 [18.00, 45.00]	28.00 [18.00, 46.00]
**Maternal religious affiliation**			
Christian	305 (70.6%)	170 (76.2%)	475 (72.5%)
Moslem	125 (28.9%)	53 (23.8%)	178 (27.2%)
Others	2 (0.5%)	0 (0.0%)	2 (0.3%)
Missing	0 (0.0%)	0 (0.0%)	0 (0.0%)
**Maternal level of education**			
None	4 (0.9%)	1 (0.4%)	5 (0.8%)
P1-P4	24 (5.6%)	11 (4.9%)	35 (5.3%)
P5-P7	117 (27.1%)	55 (24.7%)	172 (26.3%)
S1-S4	192 (44.4%)	76 (34.1%)	268 (40.9%)
S5-S6	35 (8.1%)	29 (13.0%)	64 (9.8%)
Tertiary or University	59 (13.7%)	51 (22.9%)	110 (16.8%)
Missing	1 (0.2%)	0 (0.0%)	1 (0.2%)
**Marital status**			
Married	406 (94.0%)	205 (91.9%)	611 (93.3%)
Single	23 (5.3%)	17 (7.6%)	40 (6.1%)
Divorced/Separated	3 (0.7%)	0 (0.0%)	3 (0.5%)
Widowed	0 (0.0%)	1 (0.4%)	1 (0.2%)
Missing	0 (0.0%)	0 (0.0%)	0 (0.0%)
**Maternal tribe**			
Northern	20 (4.6%)	9 (4.0%)	29 (4.4%)
Eastern	51 (11.8%)	26 (11.7%)	77 (11.8%)
Central	238 (55.1%)	110 (49.3%)	348 (53.1%)
Western	88 (20.4%)	59 (26.5%)	147 (22.4%)
Others	30 (6.9%)	16 (7.2%)	46 (7.0%)
Missing	5 (1.2%)	3 (1.3%)	8 (1.2%)
**Paternal tribe**			
Northern	26 (6.0%)	8 (3.6%)	34 (5.2%)
Eastern	48 (11.1%)	27 (12.1%)	75 (11.5%)
Central	260 (60.2%)	127 (57.0%)	387 (59.1%)
Western	73 (16.9%)	44 (19.7%)	117 (17.9%)
Others	24 (5.6%)	17 (7.6%)	41 (6.3%)
Missing	1 (0.2%)	0 (0.0%)	1 (0.2%)
**Smoking history**			
Never Smoked	432 (100.0%)	223 (100.0%)	655 (100.0%)
Stopped during index pregnancy	0 (0.0%)	0 (0.0%)	0 (0.0%)
Smoked during index pregnancy	0 (0.0%)	0 (0.0%)	0 (0.0%)
Missing	0 (0.0%)	0 (0.0%)	0 (0.0%)
**Consumption of alcohol during pregnancy**			
No	414 (95.8%)	201 (90.1%)	615 (93.9%)
Yes	18 (4.2%)	22 (9.9%)	40 (6.1%)
Missing	0 (0.0%)	0 (0.0%)	0 (0.0%)
**History of stroke**			
No	432 (100.0%)	222 (99.6%)	654 (99.8%)
Yes	0 (0.0%)	0 (0.0%)	0 (0.0%)
Don't Know	0 (0.0%)	1 (0.4%)	1 (0.2%)
Missing	0 (0.0%)	0 (0.0%)	0 (0.0%)
**HIV status**			
Negative	408 (94.4%)	217 (97.3%)	625 (95.4%)
Positive	24 (5.6%)	6 (2.7%)	30 (4.6%)
Missing	0 (0.0%)	0 (0.0%)	0 (0.0%)
**Family history of diabetes**			
No	376 (87.0%)	167 (74.9%)	543 (82.9%)
Yes	56 (13.0%)	56 (25.1%)	112 (17.1%)
Missing	0 (0.0%)	0 (0.0%)	0 (0.0%)
**Family history of pre-eclampsia**			
No	415 (96.1%)	220 (98.7%)	635 (96.9%)
Yes	16 (3.7%)	3 (1.3%)	19 (2.9%)
Missing	1 (0.2%)	0 (0.0%)	1 (0.2%)
**Family history of hypertension**			
No	298 (69.0%)	139 (62.3%)	437 (66.7%)
Yes	134 (31.0%)	84 (37.7%)	218 (33.3%)
Missing	0 (0.0%)	0 (0.0%)	0 (0.0%)
**Order of pregnancy (order_preg) — not used as a predictor (leakage, see Methods)**			
Mean (SD)	3.24 (1.35)	4.10 (1.80)	3.53 (1.57)
Median [Min, Max]	3.00 [2.00, 10.00]	4.00 [2.00, 10.00]	3.00 [2.00, 10.00]
**Number of pregnancies carried beyond 7 months**			
Mean (SD)	2.23 (1.35)	1.75 (1.64)	2.07 (1.47)
Median [Min, Max]	2.00 [1.00, 9.00]	1.00 [0.00, 7.00]	2.00 [0.00, 9.00]
**Ever delivered a baby of weight < 2.5 kg?**			
No	380 (88.0%)	194 (87.0%)	574 (87.6%)
Yes	52 (12.0%)	28 (12.6%)	80 (12.2%)
Missing	0 (0.0%)	1 (0.4%)	1 (0.2%)
**Ever delivered a baby of weight > 4kg?**			
No	354 (81.9%)	194 (87.0%)	548 (83.7%)
Yes	78 (18.1%)	26 (11.7%)	104 (15.9%)
Missing	0 (0.0%)	3 (1.3%)	3 (0.5%)
**Ever diagnosed with hypertension in a previous pregnancy?**			
No	389 (90.0%)	200 (89.7%)	589 (89.9%)
Yes	42 (9.7%)	20 (9.0%)	62 (9.5%)
Missing	1 (0.2%)	3 (1.3%)	4 (0.6%)
**History of infertility**			
No	411 (95.1%)	212 (95.1%)	623 (95.1%)
Yes	15 (3.5%)	10 (4.5%)	25 (3.8%)
Missing	6 (1.4%)	1 (0.4%)	7 (1.1%)

Corrected multigravidae-only population, *N* = 655.

### Model comparison

Random Forest achieved the highest discrimination among the three models evaluated (MCC 0.36, 95% CI 0.25–0.47; ROC-AUC 0.74, 95% CI 0.63–0.83), followed by CatBoost (MCC 0.35, 95% CI 0.18–0.46; ROC-AUC 0.71, 95% CI 0.61–0.77) (Table 2). The confidence intervals for these two models overlap substantially, indicating no statistically significant difference in performance between them. Both ensemble models outperformed the logistic-regression baseline (MCC 0.22, 95% CI 0.08–0.35; ROC-AUC 0.65, 95% CI 0.57–0.74). That both cross-validation and bootstrap validation, and all three model types, converge on a similar picture of modest-but-real discrimination serves as a sensitivity check on the robustness of these findings to modeling choice and validation method.

**Table 2:** Model comparison (repeated stratified 5-fold cross-validation, 5 repeats).

Model	MCC (95% CI)	ROC-AUC (95% CI)	Precision	Recall
Random Forest	0.36 (0.25–0.47)	0.74 (0.63–0.83)	0.61	0.50
CatBoost	0.35 (0.18–0.46)	0.71 (0.61–0.77)	0.62	0.48
Logistic Regression (baseline)	0.22 (0.08–0.35)	0.65 (0.57–0.74)	0.46	0.59

### ROC and precision-recall curves

Because only about a third of women in this population have the outcome, the precision-recall curve alongside each ROC curve is the more informative view of how many flagged high-risk predictions are actually correct (Figure 1).

**Figure 1:**
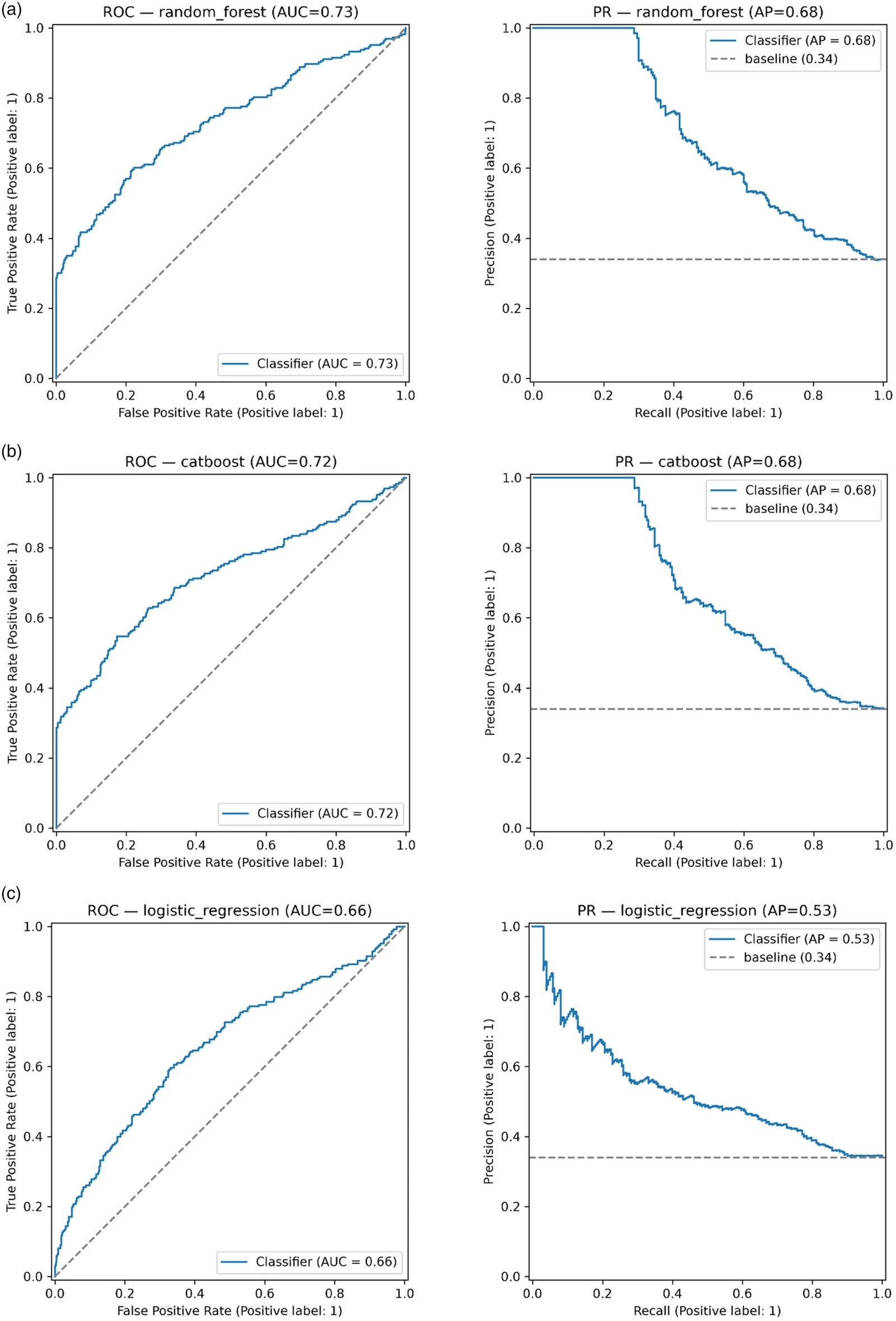
**(a)** ROC and precision-recall curves for random forest, built from out-of-fold predictions. The ROC curve (left) reaches a mean AUC of 0.74 (95% CI 0.63–0.83): a randomly selected woman with a documented history of pregnancy loss is ranked above a randomly selected woman without one about 74% of the time. The precision-recall curve (right) reaches a PR-AUC of 0.68, well above the 0.34 baseline expected from chance alone at this event rate. **(b)** ROC and precision-recall curves for CatBoost. Both curves closely track Random Forest's shape and area (ROC-AUC 0.71, 95% CI 0.61–0.77; PR-AUC 0.66), consistent with the overlapping confidence intervals in Table 1. **(c)** ROC and precision-recall curves for the logistic-regression baseline (ROC-AUC 0.65, 95% CI 0.57–0.74; PR-AUC 0.53), shown for comparison against the two ensemble models above.

### Calibration

Calibration assesses whether predicted probabilities correspond to observed event rates (Figure 2).

**Figure 2:**
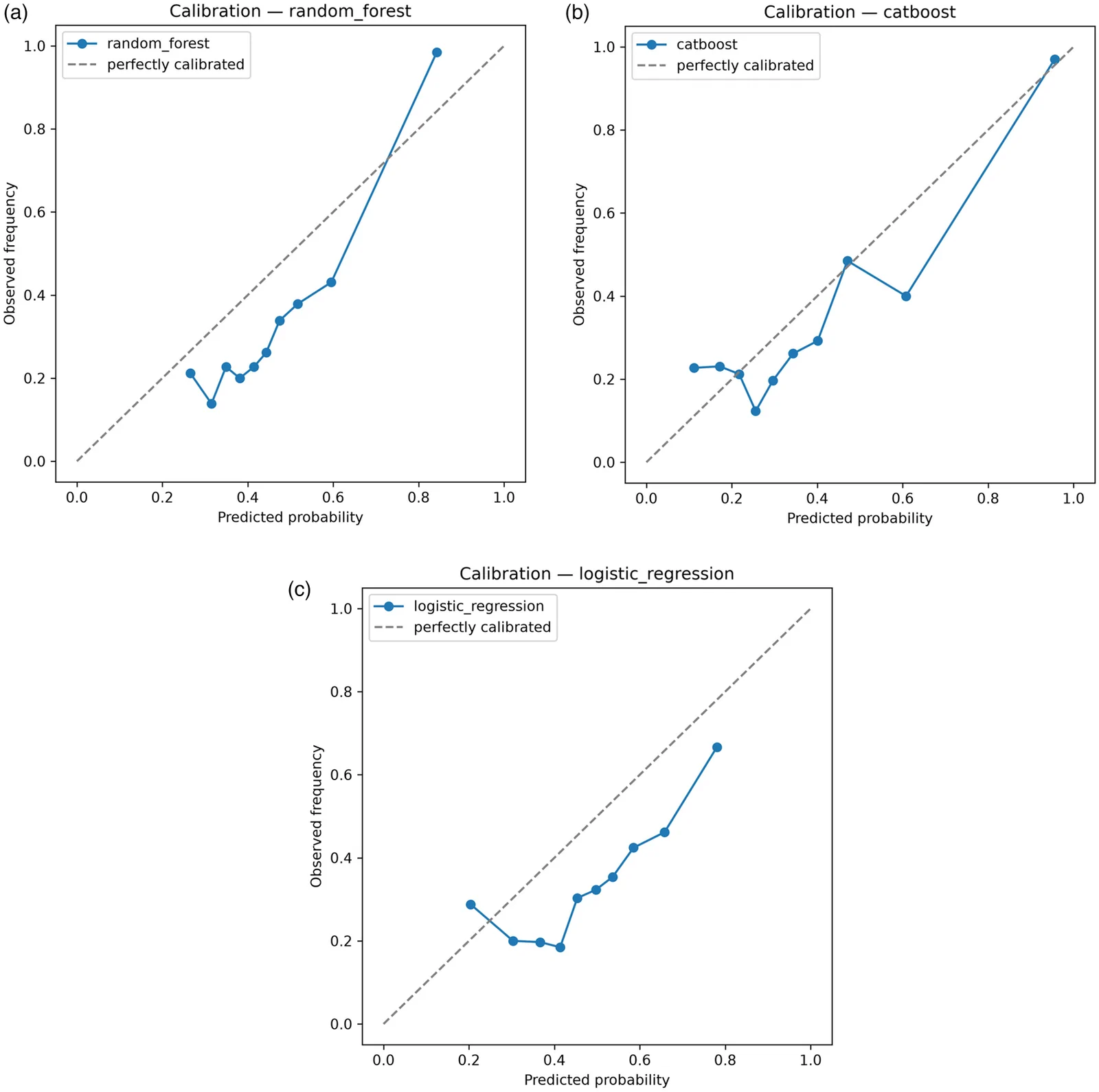
**(a)** Calibration curve for random forest. Across most of the predicted-risk range (roughly 0.25 to 0.6), the model's points sit below the diagonal, indicating a modest overstatement of risk; for example, women with a predicted risk near 0.6 had an observed event rate closer to 0.43. At the top of the risk range, the small group of women with the highest predicted risk (around 0.85) had an observed event rate near 0.98. **(b)** Calibration curve for CatBoost, showing a broadly similar pattern of modest overstatement of risk across the mid-range of predicted probabilities. **(c)** Calibration curve for the logistic-regression baseline, shown for comparison.

### Confusion matrices

Confusion matrices (Figure 3), built from out-of-fold predictions at the default 0.5 decision threshold, show the counts underlying the summary metrics in Table 1.

**Figure 3:**
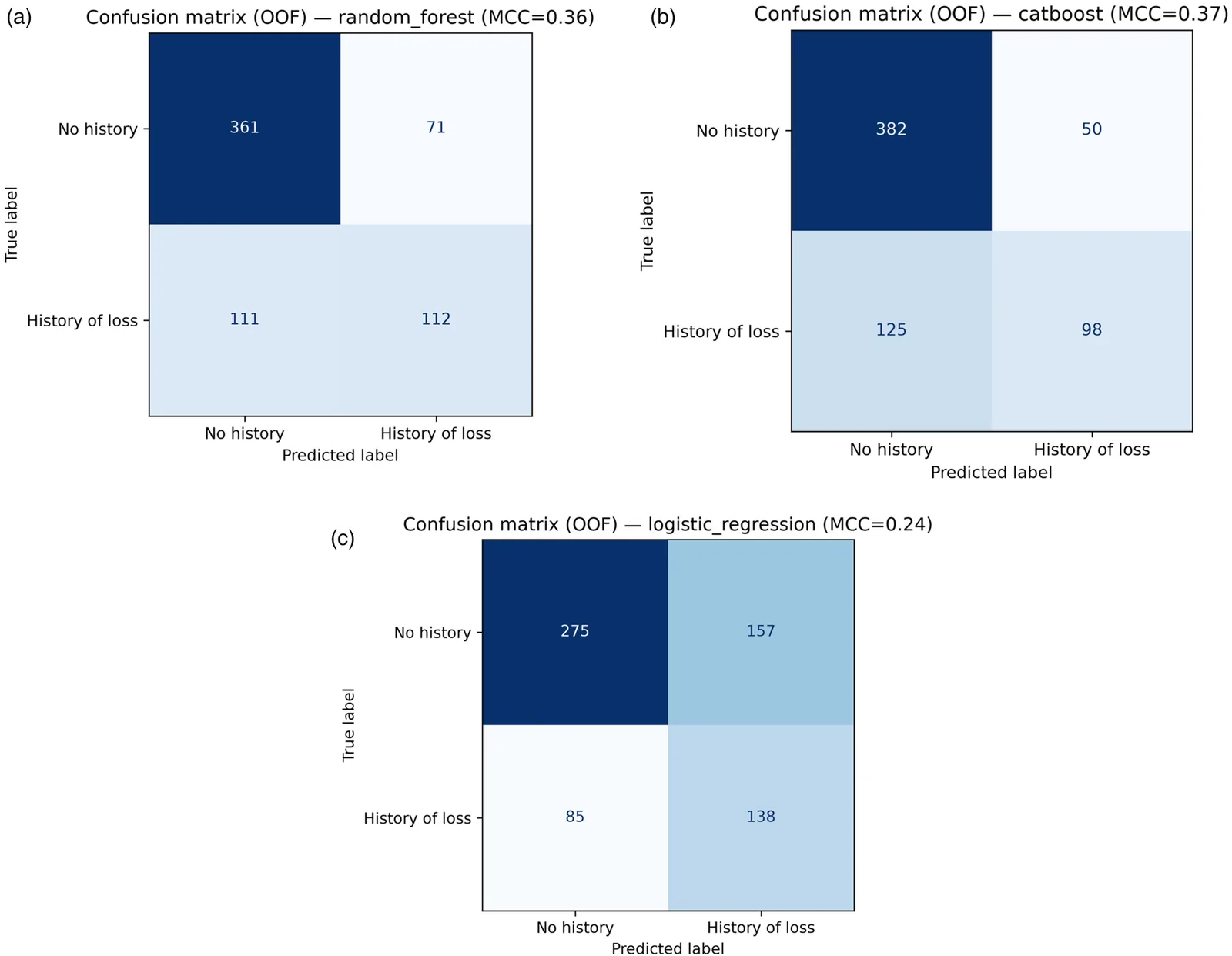
**(a)** Confusion matrix for random forest across all 655 women. Of 432 women with no documented history of pregnancy loss, 361 were correctly classified and 71 were incorrectly flagged. Of 223 women with a documented history of loss, 112 were correctly identified and 111 were missed, corresponding to a precision of 0.61 and a recall of 0.50, matching Table 1. **(b)** Confusion matrix for CatBoost, showing a similar overall pattern to Random Forest, with slightly more false negatives and slightly fewer false positives. **(c)** Confusion matrix for the logistic-regression baseline, showing more false positives and fewer false negatives than the two ensemble models, consistent with its higher recall (0.59) and lower precision (0.46).

### Decision curve analysis

Decision curve analysis quantifies whether using the model to guide a clinical decision, such as flagging a woman for closer follow-up, provides greater net benefit than a treat-everyone or treat-no-one default, across a range of threshold probabilities (Figure 4). We present this as an appropriately caveated first look at potential net benefit, not as a claim that any of these models is ready for clinical deployment; that would require prospective and externally validated evidence, which this retrospective analysis does not provide.

**Figure 4:**
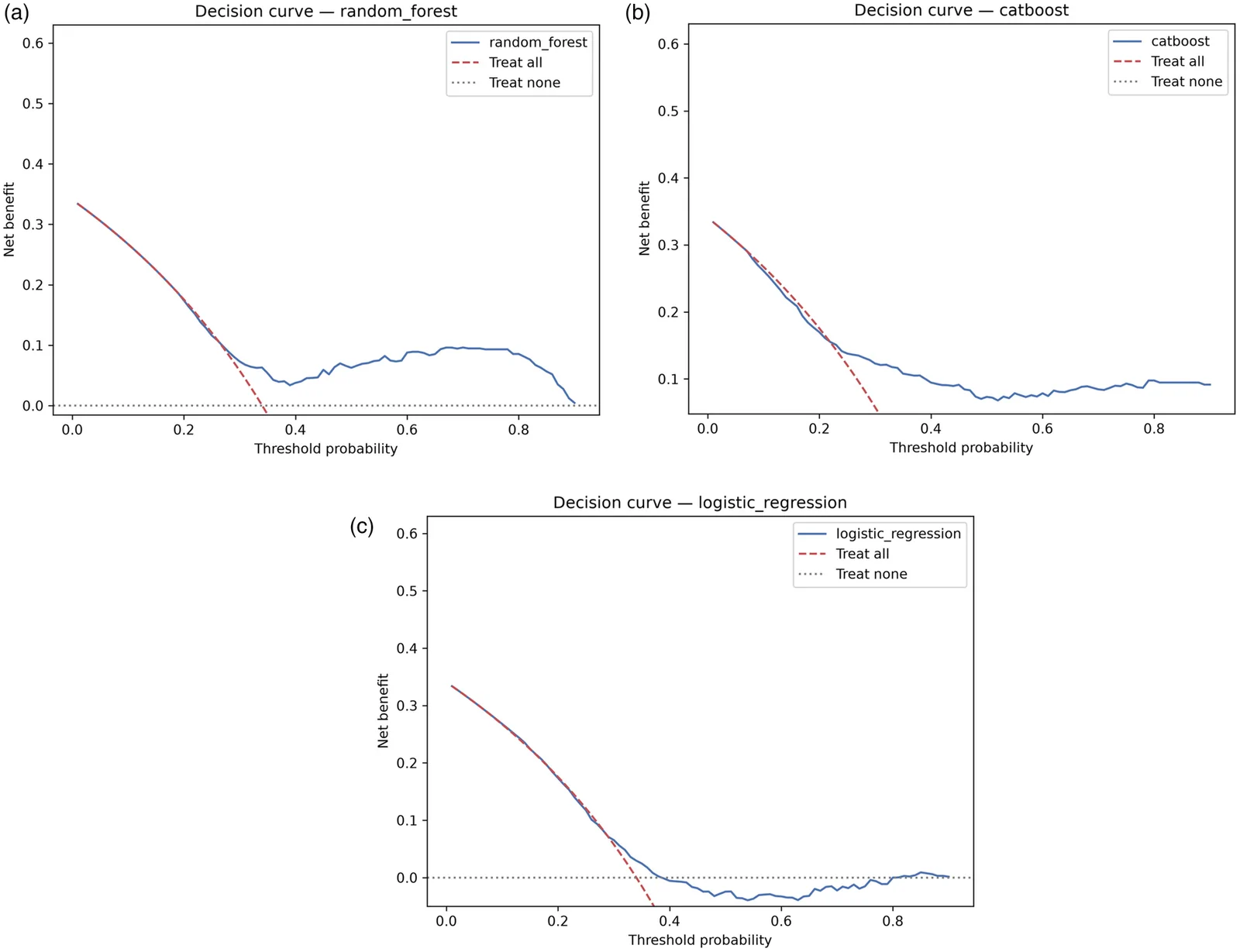
**(a)** Decision curve analysis for random forest. Below a threshold of about 0.3, net benefit tracks the treat-everyone line closely. Above 0.3, treating everyone becomes net-harmful, while the model continues to provide a small but consistently positive net benefit through a threshold of about 0.85. **(b)** Decision curve analysis for CatBoost, showing a comparable pattern of positive net benefit above a threshold of roughly 0.3. **(c)** Decision curve analysis for the logistic-regression baseline, shown for comparison.

### Predictor importance

Across both tree-based models and both importance methods, No_preg (number of pregnancies carried past seven months) ranked first, followed by Age and Education (Figure 5).

**Figure 5:**
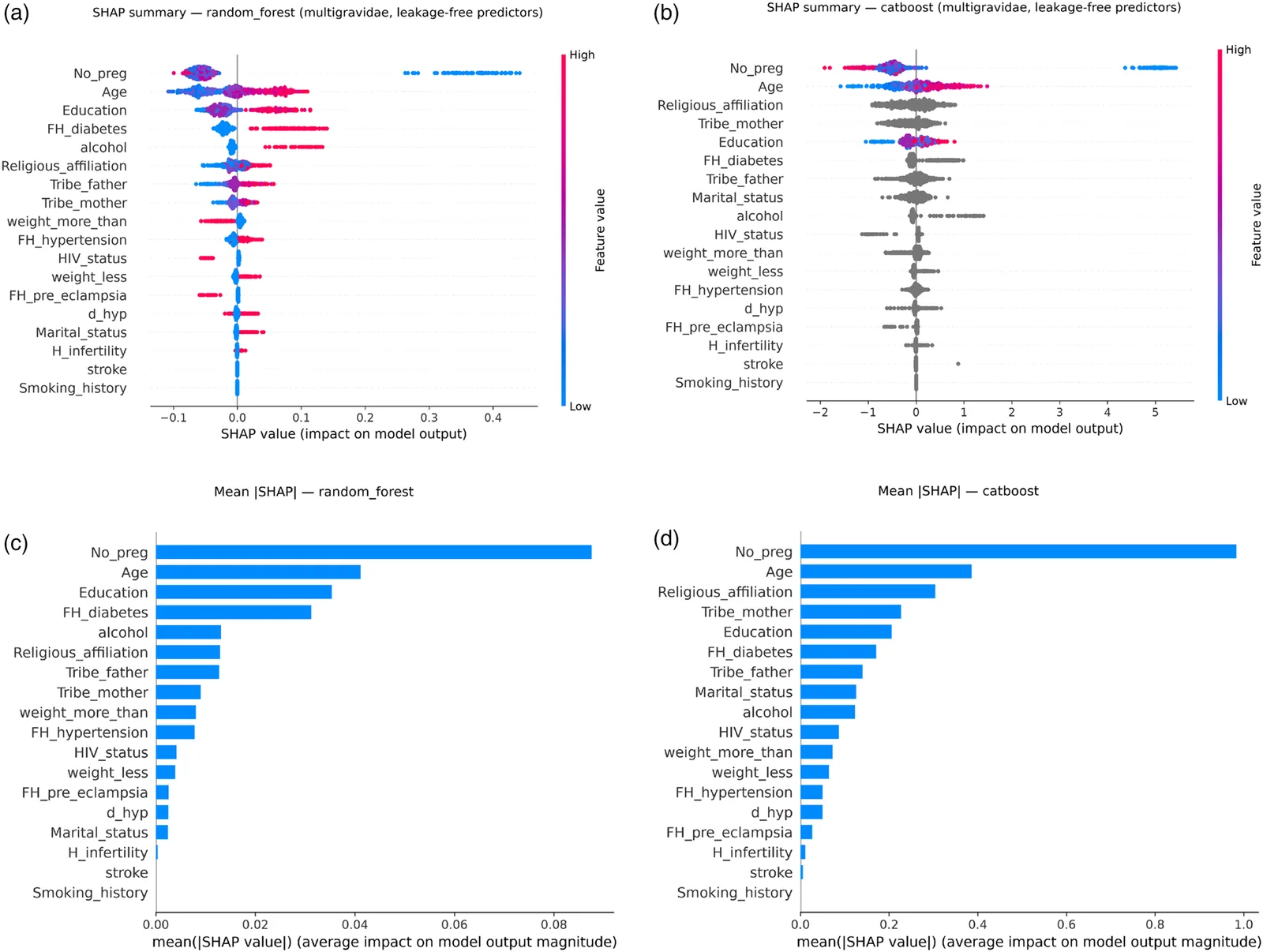
**(a)** SHAP summary plot for random forest. Each row is one predictor, ranked from most to least influential; each dot represents one of the 655 women, positioned by how much that predictor moved her individual prediction, colored by whether her value was high (red) or low (blue). No_preg is the leading predictor by a wide margin, followed by Age, Education, and family history of diabetes. Higher values of No_preg move predictions toward higher risk. **(b)** SHAP summary plot for CatBoost. The ranking mirrors Random Forest closely, with No_preg first, followed by Age, Religious_affiliation, Tribe_mother, and Education. **(C)** Mean absolute SHAP value per predictor for Random Forest: No_preg (0.088), Age (0.041), Education (0.035), and family history of diabetes (0.031). **(d)** Mean absolute SHAP value per predictor for CatBoost: No_preg (0.98), Age (0.39), Religious_affiliation (0.30), and Tribe_mother (0.23). Magnitudes are not directly comparable across model types; the ordering matches Figure 5c.

Permutation importance, an independent cross-check measuring the drop in cross-validated performance when a predictor's values are randomly shuffled, corroborated this ordering (Figure 6).

**Figure 6:**
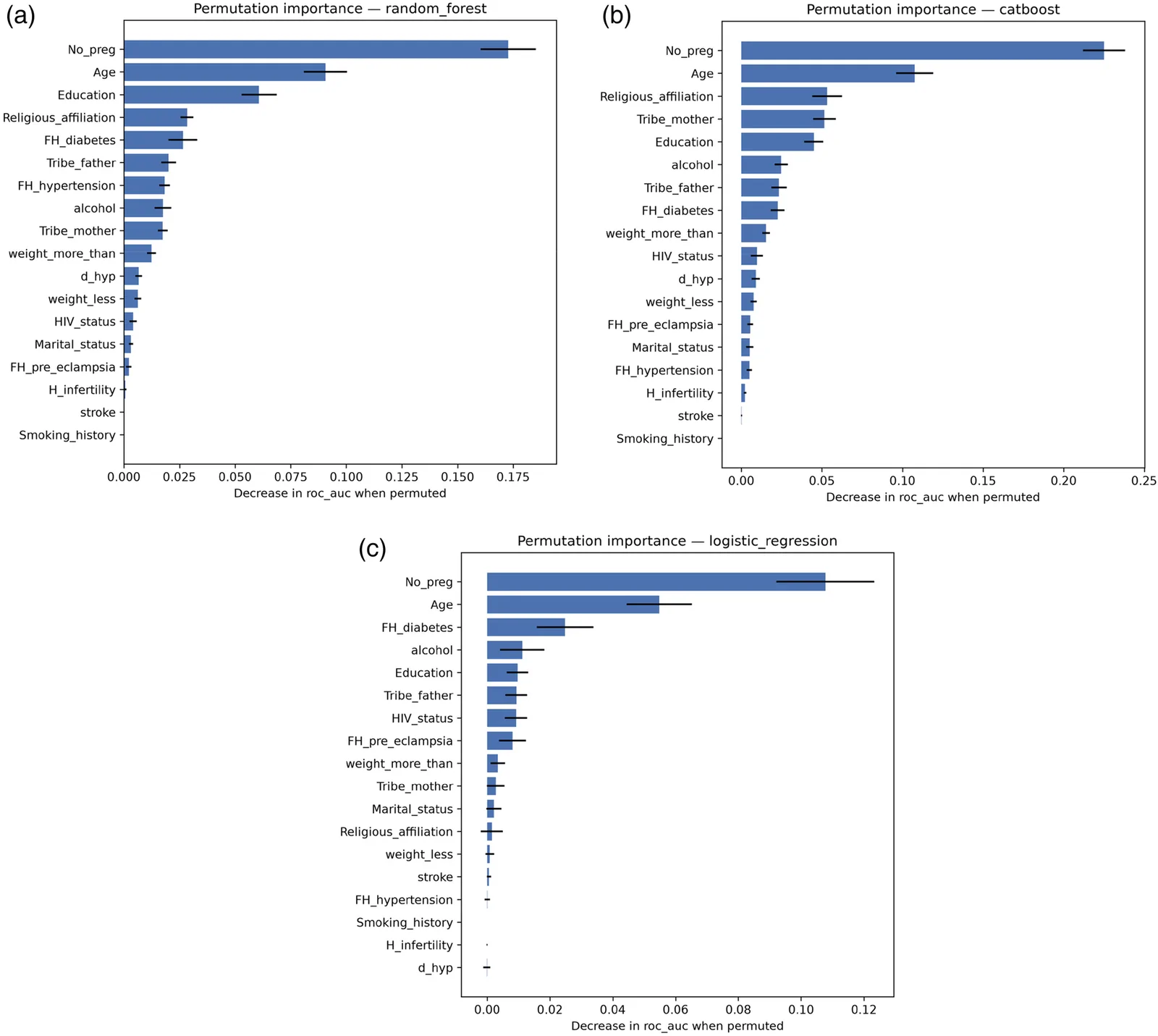
**(a)** Permutation importance for random forest. No_preg produces the largest drop in performance (0.173), followed by Age (0.091) and Education (0.061), matching the ordering found by SHAP. **(b)** Permutation importance for CatBoost: No_preg (0.225), Age (0.108), and Religious_affiliation (0.053), matching the SHAP-based ordering for this model. **(c)** Permutation importance for the logistic-regression baseline. SHAP explanations of this kind are not defined for a linear model, so permutation importance is the comparable view here. No_preg and Age remain the two leading predictors.

### Illustrative composite profiles

To help ground the predictor-importance results above, we describe two composite profiles synthesized from the models' aggregate SHAP and permutation-importance patterns. These are illustrative composites built from group-level model output, not records of any individual woman in the dataset, and are not diagnostic case descriptions.

Composite profile A (lower model-predicted association): a woman in her mid-twenties, with tertiary education, one or two prior pregnancies carried past seven months, and no reported family history of diabetes or hypertension. Across both tree-based models, this combination of predictor values was associated with a lower model-predicted likelihood of a documented history of pregnancy loss.

Composite profile B (higher model-predicted association): a woman in her mid-thirties or older, with three or more prior pregnancies carried past seven months, primary-level education only, and a reported family history of diabetes or hypertension. Across both tree-based models, this combination was associated with a higher model-predicted likelihood of a documented history of pregnancy loss. These composites illustrate the direction of the leading correlates shown in Figures 5, 6; individually, none of these factors is diagnostic, and the underlying associations are correlational rather than causal, as discussed further below.

### Subgroup performance

Model discrimination was broadly, though not perfectly, consistent across reliable subgroups defined by maternal age (Figure 7), education (Figure 8), and tribal region (Figure 9).

**Figure 7:**
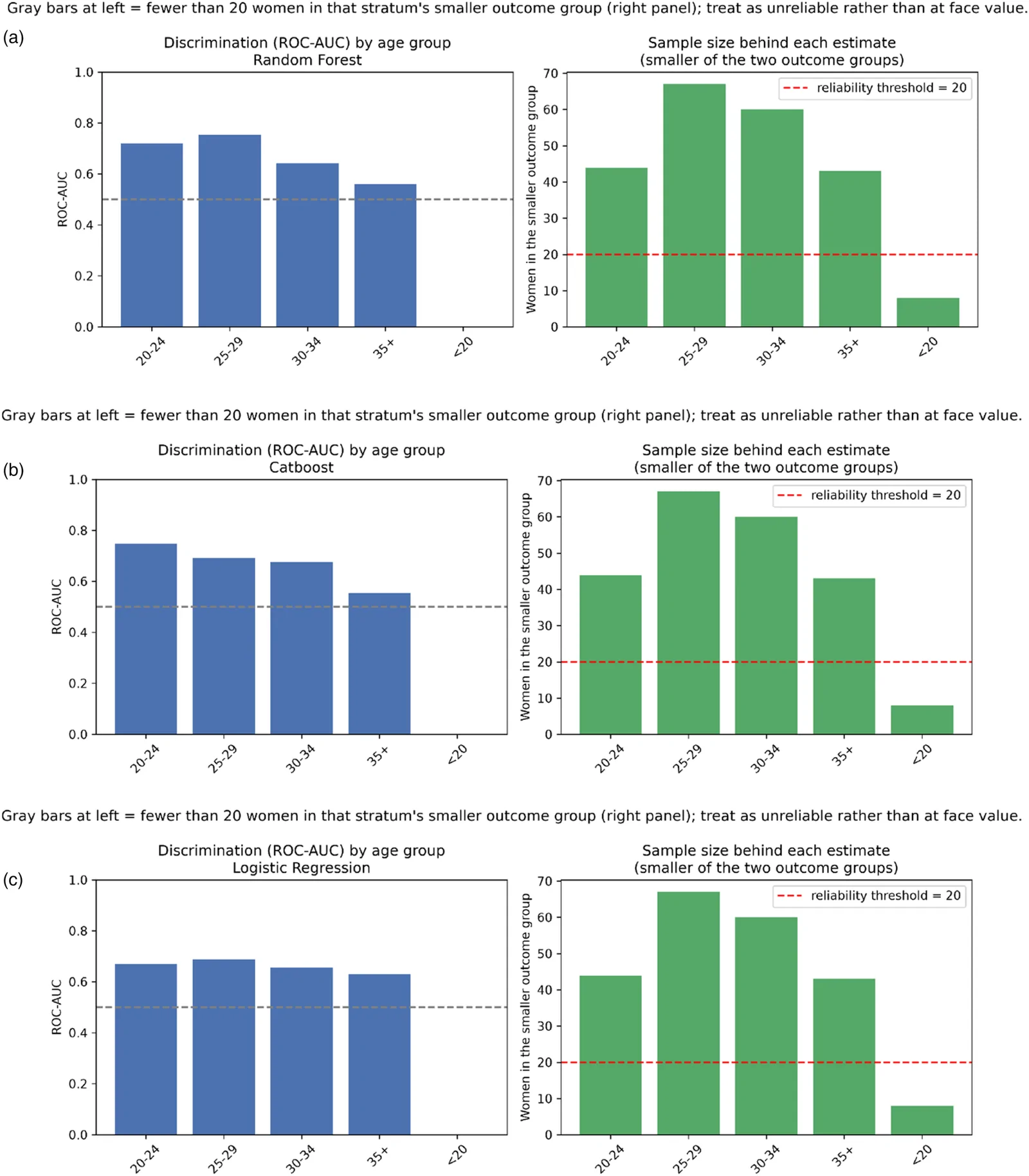
**(a)** Random forest ROC-AUC by maternal age group (left) and the number of women in each group's smaller outcome group (right), with the dashed red line marking the 20-woman reliability threshold. Four of five age bands (20–24, 25–29, 30–34, 35+) met the threshold, with ROC-AUC ranging from 0.56 in women 35 and older to 0.75 in women 25 to 29. Women under 20 (*n* = 17; 8 in the smaller outcome group) did not meet the threshold and are shown in gray. **(b)** CatBoost ROC-AUC by maternal age group, showing the same reliability pattern. **(c)** Logistic-regression ROC-AUC by maternal age group, shown for comparison.

**Figure 8:**
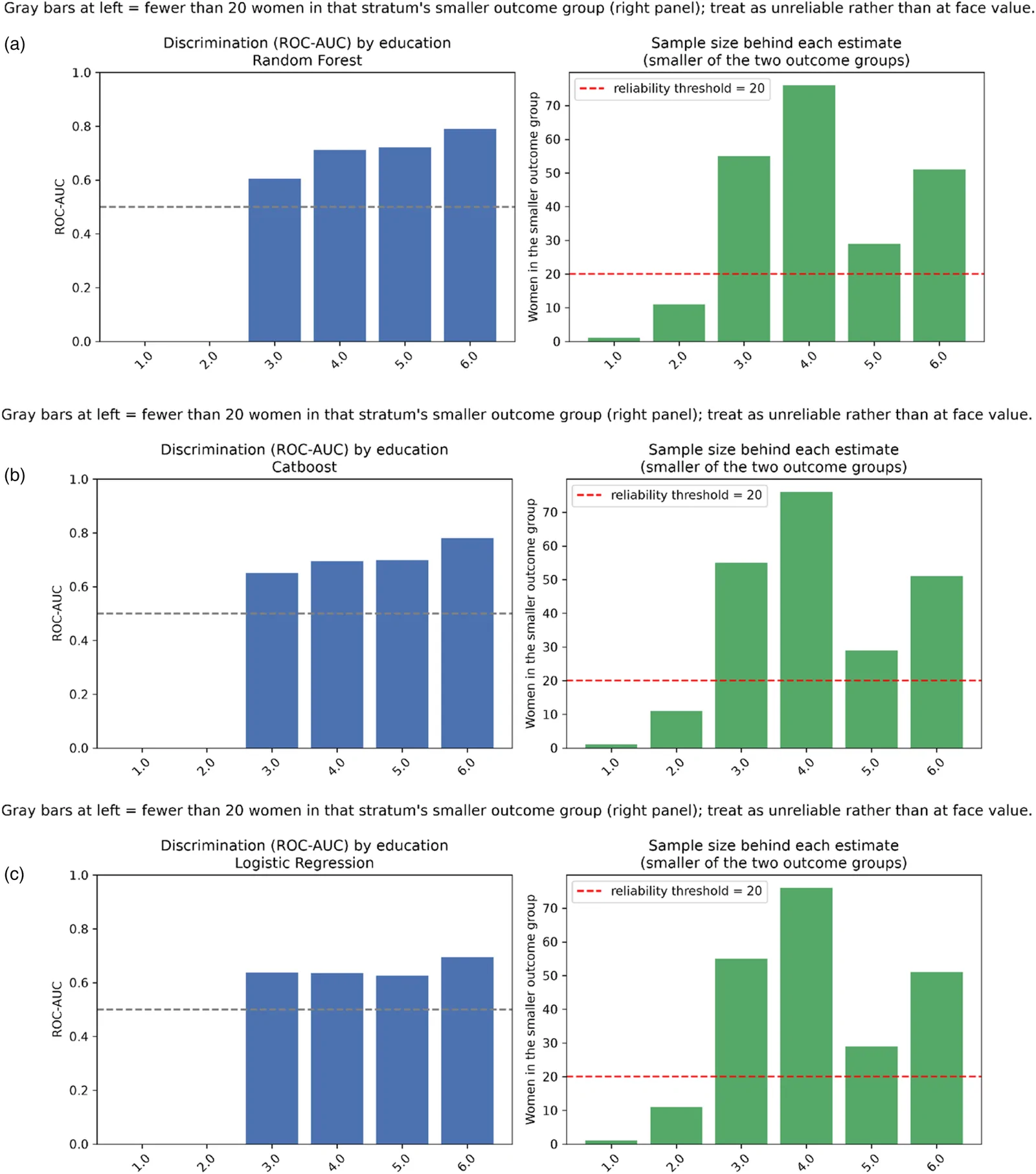
**(a)** Random forest ROC-AUC by education level (left) and sample size (right). Four of six education categories met the 20-woman reliability threshold, with ROC-AUC ranging from 0.61 to 0.79. The two smallest categories (each under 40 women) did not meet the threshold and are shown in gray. **(b)** CatBoost ROC-AUC by education level, showing a broadly similar reliability pattern. **(c)** Logistic-regression ROC-AUC by education level, shown for comparison.

**Figure 9:**
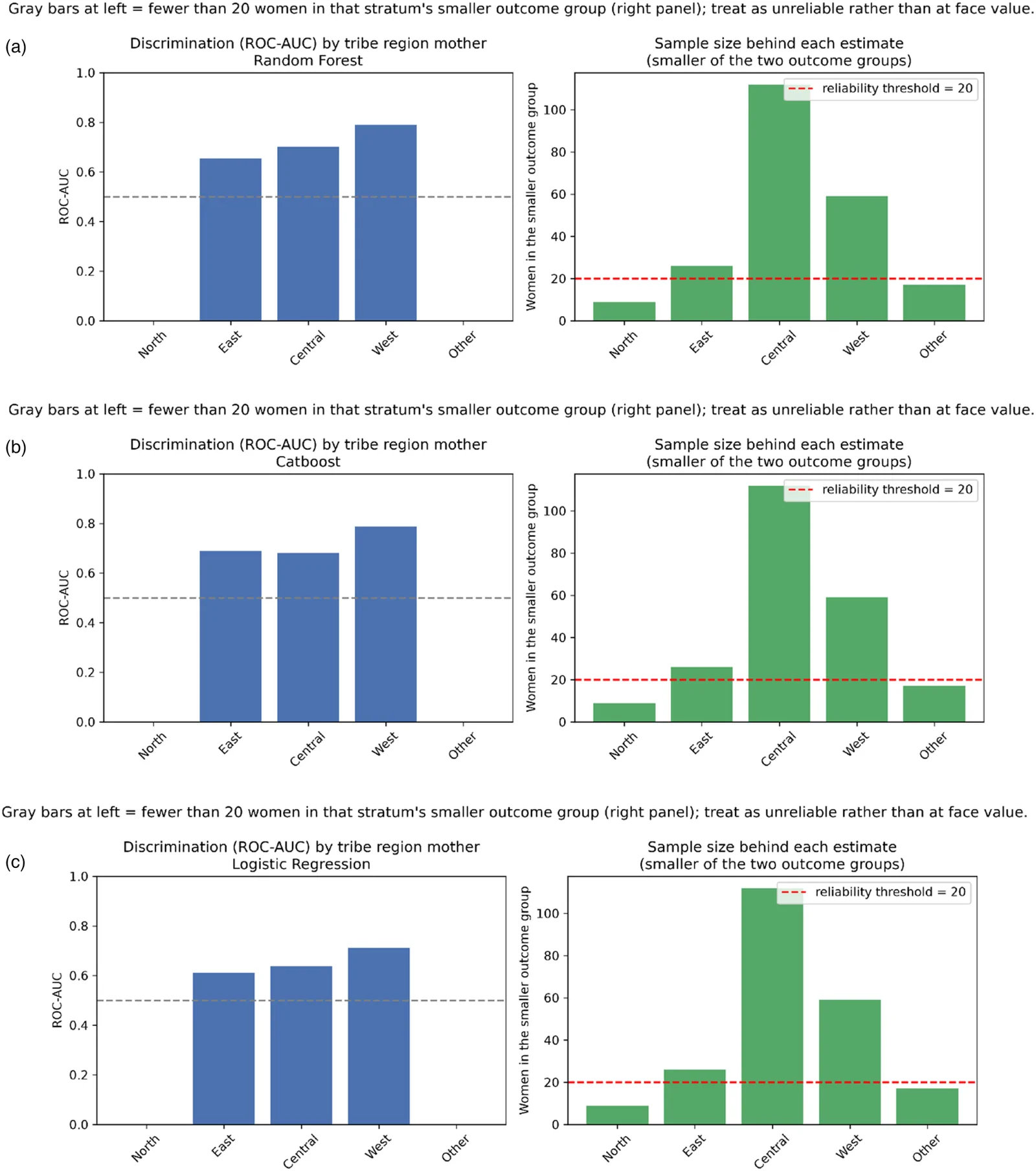
**(a)** Random forest ROC-AUC by maternal tribal region (left) and sample size (right). **(b)** CatBoost ROC-AUC by maternal tribal region, showing the same three reliable regions and a comparable range of values. **(c)** Logistic-regression ROC-AUC by maternal tribal region, shown for comparison.

Three of five maternal tribal regions (East, *n* = 77; Central, *n* = 352; West, *n* = 148) met the reliability threshold, with ROC-AUC ranging from 0.65 to 0.79 across those three. The remaining two regions (North, *n* = 31; Other, *n* = 47) did not meet the threshold and are reported as unreliable. None of the three reliable regions showed a substantially different ROC-AUC from the others.

## Discussion

This study set out to identify sociodemographic, medical-history, and obstetric-history factors associated with a documented history of pregnancy loss among multigravid women in an indigenous African population. Random Forest and CatBoost showed comparable, modest discrimination (MCC 0.36 and 0.35, respectively, with overlapping confidence intervals), both outperforming a logistic-regression baseline (MCC 0.22), consistent with evidence that ensemble tree methods offer a real but incremental advantage over regression once validation bias is properly controlled (13).

In constructing these models, we utilized a rigorous data preprocessing and feature selection process, to include a range of demographic, socio-economic, and health-related variables. The leading correlates identified, including number of pregnancies, maternal age, maternal level of education, and a family history of diabetes, have been described in previous maternal health literature (22–25). In SSA, demographic and health survey-based studies (26, 27), and a Rwandan cohort (28)also converge on similar demographic and access-related variables. Furthermore, a large prospective preconception cohort in North America (29)and a systematic review of 22 recurrent pregnancy loss models (30)both identify maternal age and pregnancy-loss history as the two most consistently important variables across very different study designs and populations. This convergence lends some external validity to the associations found here, even though the outcome definitions, study designs, and populations differ across studies.

However, we are cautious about how these associations should be interpreted, particularly for tribe and religious affiliation. Both may be interpreted as non-causal proxies for socioeconomic status, geography, and differential access to healthcare, rather than as biological or genetic risk factors in their own right. Specific tribal regions may differ in exposure to endemic disease, nutritional deficiency (31), or access to healthcare, any of which could plausibly explain an association with pregnancy-loss history without implying a direct biological pathway. Treating these group-level socioeconomic proxies as though they carry causal or biological meaning risks reinforcing the very kind of algorithmic unfairness that has been a concern in machine learning in health care more broadly (32).

Our study's use of ML to identify factors associated with pregnancy loss contributes to a growing body of research globally applying advanced analytical techniques to maternal and child health challenges. Current literature has documented the use of various ML models in predicting adverse pregnancy outcomes, although none of these studies have utilized data from SSA. A study by (33)utilized XGBoost, among other models, to predict miscarriage in patients with immune abnormalities, achieving an impressive AUC of 0.9209, highlighting the potential of the use of ML algorithms in enhancing predictive accuracies in obstetrics (33). Another multi-center study in China explored the use of a number of ML models to predict threatened miscarriage based on hormonal markers such as AEA, progesterone, and *β*-hCG. This study underscored the relevance of integrating biological markers with ML to improve prediction models, a methodological approach that resonates with the variable selection in our study (34). However, both these studies had models with significantly improved performance compared to the current study. It is important to note that models developed for such specific groups (that is, immune and medication-related variables in women at increased risk of adverse outcomes and threatened abortion using hormonal markers) utilizing detailed, relevant clinical data, leading to higher predictive performance (AUC). In contrast, the current study's broader and more varied population included a wider range of risk factors and outcomes, making it inherently more challenging to achieve a high AUC.

In the current study, we identified maternal age, number of previous pregnancies carried beyond 7 months, maternal education level, ancestral heritage, and a family history of diabetes and hypertension as the most important variables in predicting miscarriage. This selection list is supported by previous literature, which consistently shows that these factors are significantly associated with the risk of miscarriage. For instance, increasing maternal age has been linked to a higher risk of pregnancy loss, especially as women age beyond 35 years (35). This is compounded if the paternal age is over 40, highlighting the impact of parental ages on pregnancy outcomes (36). However, the parent study did not collect data on the partner's age, and we recommend its inclusion in future work in African populations. Additionally, a history of multiple pregnancies, particularly previous miscarriages, is known to increase the risk of subsequent miscarriages (37), indicating the importance of pregnancy history in predicting outcomes. Particularly, the risk of having a miscarriage is increased with the number of previous pregnancies but decreases with the number of previous live births. In the current study, we did not document how many of the previous pregnancies were live births versus miscarriages. Additionally, it should be considered that the relationship between number of pregnancies and one's risk of miscarriages may be confounded by age, which was also identified to be a strong risk factor.

While the influence of the level of education and ancestral heritage (the tribe of father and mother) on miscarriage risk is less directly studied, these factors are known to interact with health behaviors and access to care, which can indirectly affect one's risk of miscarriage. Based on ancestral heritage, specific tribes may have a higher prevalence of certain hereditary conditions that can affect pregnancy outcomes. Previous comparisons between tribes have found the risk of pregnancy loss to be different between certain tribes in Uganda (38). Environmentally, tribal regions might expose members to particular health risks, such as endemic diseases or nutritional deficiencies that could impact maternal health. For example, a study examining mother-child dyads across 25 SSA (31) highlighted the extensive prevalence and impact of nutritional deficiencies, affecting both mothers and children, pointing out the increasing vulnerability to infections and reduced overall resilience, potentially leading to higher miscarriage rates in women from such regions. We are however cautious about how these associations should be interpreted. One's tribe or ancestral heritage may be interpreted as non-causal proxies for socioeconomic status, geography, and differential access to healthcare, rather than as biological or genetic risk factors in their own right. Specific tribal regions may differ in exposure to endemic disease, nutritional deficiency (31), or access to healthcare, any of which could plausibly explain an association with pregnancy-loss history without implying a direct biological pathway. Treating these group-level socioeconomic proxies as though they carry causal or biological meaning risks reinforcing the very kind of algorithmic unfairness that has been a concern in machine learning in health care more broadly (32).

On the other hand, the level of education significantly impacts the risk of miscarriage by influencing health knowledge, behaviors, access to healthcare, stress management, and work environments. Higher educational attainment generally leads to a better understanding and management of health during pregnancy, promoting behaviors that may reduce one's risk for pregnancy loss, such as maintaining proper nutrition and avoiding harmful substances. Moreover, educated individuals tend to have better access to quality healthcare services, which enhances prenatal care and early intervention for potential complications (39).

Conditions such as diabetes and hypertension, family history of which was found to be important in the current risk prediction model, are directly linked to vascular health (40), potentially impacting pregnancy viability and increasing the risk of complications such as miscarriage. Both conditions have a strong genetic predisposition and may also point towards one's lifestyle, increasing the risk of developing such conditions or their existence before diagnosis. However, in the current study, we did not establish the existence of these conditions in any of the respondents; we solely relied on self-reported family health history reported by participants, which can be subject to reporting bias. Future work should consider including additional variables pertaining to the specific patient's medical history to improve performance and accuracy.

This study has several strengths. It is among the first studies to utilize data from an indigenous African population to develop a prognostic ML model for assessing factors associated with pregnancy loss among women of childbearing age. This study utilizes a dataset with many demographic, socio-economic, and health-related variables, providing clinical and medical factors and socio-demographic factors that influence miscarriage risk. Second, the use of model validation techniques, including cross-validation and the application of feature importance analysis through SHAP values, provides transparency and clarity on the predictive contributions of each variable. The current work provides internally validated, appropriately caveated set of candidate factors and an evaluation pipeline (multi-model comparison, repeated cross-validation with bootstrap cross-checking, calibration, and subgroup reliability reporting) that can guide the design of future prospective studies that follow women from a defined point in pregnancy through to a directly observed outcome, ideally incorporating partner age (36) and a clearer separation of miscarriage, induced abortion, and ectopic pregnancy as distinct outcomes.

### Limitations

First, the outcome is a retrospective, self-reported history of pregnancy loss. This means the associations reported here describe correlates of having ever experienced pregnancy loss, and should not be read as predicting whether a woman's current or future pregnancy loss. Second, we combined miscarriage, induced abortion, and ectopic pregnancy into a single outcome because all three share the same defining endpoint (the pregnancy does not continue to a live birth), consistent with how pregnancy loss has been analyzed in prior work in this setting (2); ectopic pregnancies were also too few in this sample to model separately. Nonetheless, we are aware that the specific biological mechanisms and risk factors underlying these three events plausibly differ, and pooling them may obscure outcome-specific associations that a larger, purpose-designed study could describe. Third, no external validation was performed; the repeated cross-validation and bootstrap comparison reported here, and the consistency of findings across three model types, provide some reassurance about internal robustness but cannot substitute for validation in an independent population, which we identify as a priority for future work.

Fourth, several socioeconomic variables, including occupation and financial status, were self-reported and may be subject to reporting bias. Reporting calibration, decision curve analysis, subgroup reliability, and a structured comparison against six published studies addresses reporting gaps that a recent systematic review identified as common in this literature (41). Finally, this study only included 655 multigravida women in the analysis. Nevertheless, the factors associated with a history of pregnancy loss identified in the present study have also been reported in previous literature (22), lending support to the plausibility of our findings and reinforcing the hypothesis-generating nature of this work.

Future research should, therefore, consider the following recommendations to advance the use of ML models in predicting the risk of pregnancy loss and other adverse pregnancy outcomes. First, as we have outlined previously, there's a need to consider biochemical or biophysical markers of pregnancy loss risk and a more granular etiological classification of cases (endocrine, immunological, genetic, thrombophilic, or anatomic), which the sociodemographic and historical variables available here cannot provide but which future prospective studies, ideally combining clinical and laboratory data with the sociodemographic factors examined here, could incorporate. Secondly, external validation of such models across different geographical and ethnic populations in sSA would ensure its generalizability and applicability in diverse settings. Such studies could help refine the model's predictive accuracy and adapt it to various cultural and healthcare contexts. There's also the need to establish contextually relevant evidence on the acceptability and feasibility of health workers and other relevant stakeholders to utilize such models in clinical practice. Understanding such factors beforehand can significantly mitigate potential fears and allow seamless integration of such data-driven approaches in resource-limited healthcare settings. Finally, ongoing research should also focus on the ethical aspects of implementing AI in healthcare, particularly concerning issues of privacy, data security, and the potential for bias in automated decision-making processes. Ensuring these models are transparent and equitable will be essential for their acceptance and effectiveness in clinical settings.

## Conclusions

In this retrospective analysis of 655 multigravid women in an indigenous African population, several sociodemographic and obstetric-history factors (number of pregnancies carried past seven months, maternal age, and maternal education) were statistically associated with a documented history of pregnancy loss, with broadly consistent results across two ensemble machine-learning models, two independent validation methods, and two predictor-importance methods. Because the outcome reflects reproductive history rather than the result of a directly observed pregnancy, these findings should be read as hypothesis-generating: they identify candidate factors and an evaluation framework that can inform the design of a future prospective, externally validated study of miscarriage risk, rather than constituting a tool ready for identifying women at risk of a future pregnancy loss.

## Data Availability

The raw data supporting the conclusions of this article will be made available by the authors, without undue reservation.
